# Multi-physics interactions drive VEGFR2 relocation on endothelial cells

**DOI:** 10.1038/s41598-017-16786-4

**Published:** 2017-12-01

**Authors:** Valentina Damioli, Alberto Salvadori, Gian Paolo Beretta, Cosetta Ravelli, Stefania Mitola

**Affiliations:** 1 Università degli Studi di Brescia, DIMI Department of Mechanical and Industrial Engineering, Brescia, 25123 Italy; 2 Università degli Studi di Brescia, DICATAM, Department of Civil, Environmental, Architectural Engineering and Mathematics, Brescia, 25123 Italy; 3 Università degli Studi di Brescia, DMMT, Department of Molecular and Translational Medicine, Brescia, 25123 Italy; 4 Laboratory for Preventive and Personalized Medicine (MPP Lab), Università degli Studi di Brescia, Brescia, 25123 Italy

## Abstract

Vascular Endothelial Growth Factor Receptor-2 (VEGFR2) is a pro-angiogenic receptor, expressed on endothelial cells (ECs). Although biochemical pathways that follow the VEGFR2 activation are well established, knowledge about the dynamics of receptors on the plasma membrane remains limited. Ligand stimulation induces the polarization of ECs and the relocation of VEGFR2, either in cell protrusions or in the basal aspect in cells plated on ligand-enriched extracellular matrix (ECM). We develop a mathematical model in order to simulate the relocation of VEGFR2 on the cell membrane during the mechanical adhesion of cells onto a ligand-enriched substrate. Co-designing the *in vitro* experiments with the simulations allows identifying three phases of the receptor dynamics, which are controlled respectively by the high chemical reaction rate, by the mechanical deformation rate, and by the diffusion of free receptors on the membrane. The identification of the laws that regulate receptor polarization opens new perspectives toward developing innovative anti-angiogenic strategies through the modulation of EC activation.

## Introduction

Tyrosine Kinase Receptors (RTKs) transmit information from the extracellular to the intracellular microenvironment and play a central role in physiological and pathological conditions, including tumor progression. RTKs catalyze the phosphorylation of Tyrosine residues in their sequences as well as in second messengers. The non-homogeneous distribution of lipids and proteins on the cell membrane, both in space and time, is highly dynamic at multiple spatial levels and orchestrates the cellular response to different biochemical and mechanical inputs^1^. Membrane dynamics and composition^2,3^ also govern the expression and the activation of Epidermal Growth Factor Receptor (EGFR) and Vascular Endothelial Growth Factor Receptor-2 (VEGFR2). The latter, expressed by cancer and ECs, modulates angiogenesis and tumor progression^4–7^ by binding different soluble ligands, including VEGF-A, the non-canonical HIV-1-Tat^8,9^, and gremlin^10,11^. Thus, an abnormal spatial regulation of RTKs may play a role in cancer progression^12^. Most of VEGFR2 ligands contain a heparin binding domain and accumulate in the ECM, supporting a long-lasting activation of the cells. Moreover, ligand-enriched ECM recruits VEGFR2 at the basal aspect of ECs^13,14^, leading to a polarization of intracellular molecules.

Receptor-ligand interactions have been extensively studied from the biological and computational point of view. Several mathematical models have been developed to describe the body distribution of different isoforms of canonical and non canonical ligands of VEGFR2 and their interactions with VEGFRs both *in vitro* and *in vivo* ^15,16^. These models confirmed that the amount of matrix-bound VEGF in normal human tissues (e.g. skeletal muscle) is 30 to 100-fold higher than the amount of free ligands^17,18^. Some models considered also receptor internalization, since similarly to other RTKs ligand interaction induces VEGFR2 endocytosis in early endosomes^19^. It is worth to point out that VEGFR2 undergoes efficiently in the endocytic compartment even in the absence of VEGF^20,21^. Mathematical models were also used to simulate and describe the competitive and/or synergic effects of different ligands on VEGFR2 interaction and biological cell responses^22^.

The goal of this study is threefold: formulating a mathematical model of VEGFR2 recruitment in EC, simulating the dynamics of VEGFR2 in EC seeded on ligand-enriched ECM, and finally co-designing experimental and numerical investigations to characterize the dynamic lateral distribution (diffusion) of VEGFR2 receptors on the plasma membrane and their interactions (reaction) with immobilized ligands. The key features of our experimental evidence on VEGFR2 relocation are well captured by a diffusion-reaction model, whereby the evolving geometry of the membrane is extremely simplified. The model is mathematically rigorous and self-consistent, in that it stems from continuity equations (for mass, energy, and entropy), standard chemical kinetics, thermodynamic restrictions, and constitutive specifications^23,24^. This sequence provides the governing equations in a strong form, stated for dimensionless unknown fields and converted in a weak form prior to the numerical approximation via the Finite Element Method. Thermodynamic parameters have been inferred from the experimental analyses and from the literature, whereas a few have been calibrated.

The present model will be further developed, to include the spreading/deformation of the cell, its focal adhesion and the evolution of the stress fibers, the internalization of the complex, the ligand competition, ECM composition, co-receptor partners, and the cell cortex structure^12,25^. Moreover, the predictive capabilities of the model will be exploited in future studies to foresee alterations of the receptor behavior induced by mutations or administration of anti-angiogenic drugs.

## Results

### Free and immobilized ligands induce VEGFR2 rearrangement on EC plasma membrane

**Figure 1:**
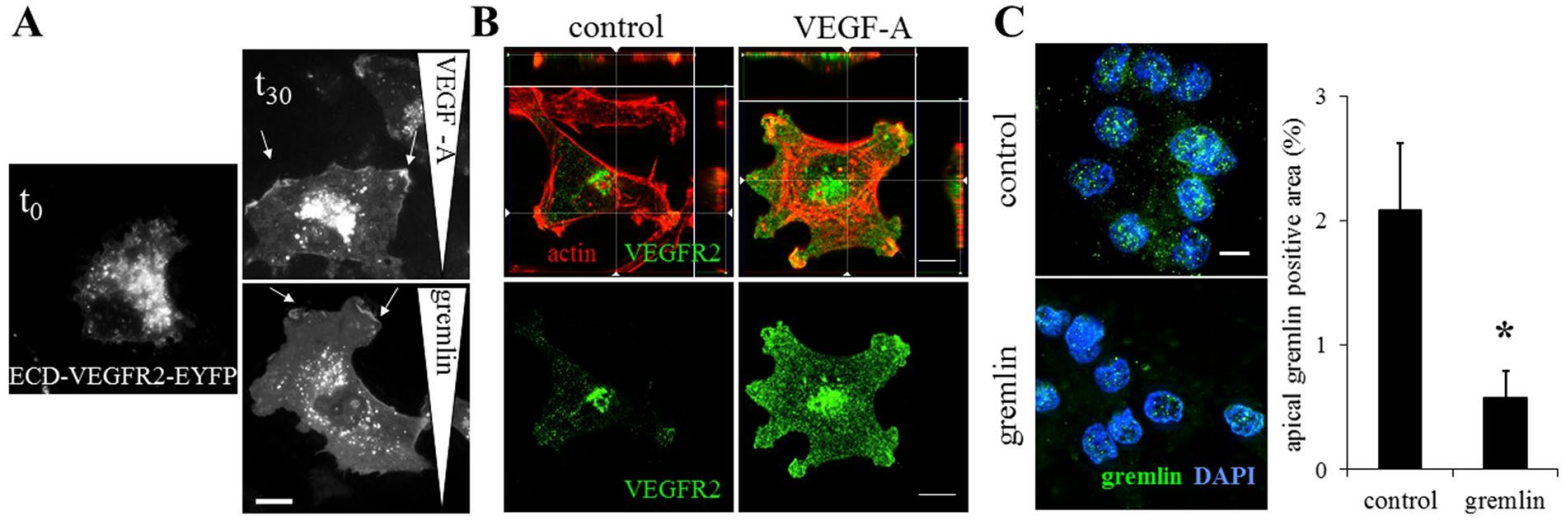
VEGF-A and gremlin induce VEGFR2 rearrangement on EC surface. (**A**) ECD-VEGFR2-EYFP ECs were stimulated by a VEGF-A or gremlin gradient for 2 hours, fixed and analysed using a Zeiss Axiovert 200M system (630×; white bar: 10 μm). Arrows indicate ECD-VEGFR2-EYFP-enriched cell lamellipodia. (**B**) HUVECs adherent on Fibrinogen or VEGF-A-enriched substrates were stained for VEGFR2 (green) and actin (red) and analysed using a LSM510 Meta confocal microscope. Images show the basal portion of adherent cells with the orthogonal *z* reconstruction of the whole cell (630×; white bar: 10 *μ*m). (**C**) VEGFR2-EC, seeded on immobilized gremlin or on coverglass for 4 hours, were incubated with 150 ng/mL of gremlin for 90 minutes at 4 °C and washed with 1.5 mol/L NaCl. VEGFR2-bound gremlin, in the apical portion of the cells, was detected by immunofluorescence analysis using a Zeiss Axiovert 200 M microscope system (630x; white bar: 10 *μ*m). Data are expressed as percentage ± s.d. of gremlin positive area with respect to the total cell area (n = 20 cells/sample; *P < 0.001, Student’s t-test).

Different canonical and non canonical ligands, including VEGF-A, HIV-1-Tat, and gremlin are able to activate VEGFR2 and induce VEGFR2-mediated EC proliferation and migration^9,10^. To assess whether VEGFR2 redistributes on the EC membrane when challenged by free ligands, adherent ECs over-expressing the Enhanced Yellow Fluorescent Protein (EYFP)-labeled extracellular domain (ECD) of VEGFR2 (ECD-VEGFR2-EYFP) were exposed for 2 hours to a linear concentration gradient of free ligands, including gremlin or VEGF-A, in a 2D chemotaxis assay. According to our previous investigations data, ECD-VEGFR2-EYFP exerts binding and dimerization activities similar to the VEGFR2 full length^10,11,14^. Figure 1A shows that ECD-VEGFR2-EYFP is equally distributed on non-treated ECs (t_0_), while the gradient of ligands induces the ECD-VEGFR2-EYFP to be recruited in the lamellipodia at the leading edge of migrated ECs (t_30_). Together, these data demonstrate that free ligands are able to induce EC polarization, leading to VEGFR2 relocation on the surface of ECs. Although VEGFR2 ligands are usually considered as soluble molecules, *in vivo* they are bound and immobilized in the ECM or on the cell membrane by heparan-sulphate proteoglycans^26^.

Soluble and ECM-bound VEGF impact VEGFR2 trafficking rate. The clustering and the slower internalization rate of VEGFR2 complexes activated by ECM-bound VEGF elicits a prolonged activation of VEGFR2 and Extracellular signal Regulated Kinase (ERK) with a different pattern of site-specific phosphorylation^14,27,28^. To characterize the influence of the immobilized VEGFR2-ligands on the VEGFR2 rearrangement on the cell membrane, we plated ECs on ligand-coated cell plates. Similarly to immobilized gremlin^14^, immobilized VEGF-A induces the recruitment of VEGFR2 to the plasma membrane at the basal aspect of ECs, thus leading to a localized and directional receptor activation (Fig. 1B). The concentration of VEGFR2 at the apical side of the cell is diminished by the recruitment of VEGFR2 at the basal portion of adherent cells, as demonstrated by the reduction of soluble ligand binding ability (Fig. 1C). Similar data were obtained with immobilized-VEGF-A.

### Ligand binding decreases VEGFR2 diffusion on plasma membrane

**Figure 2:**
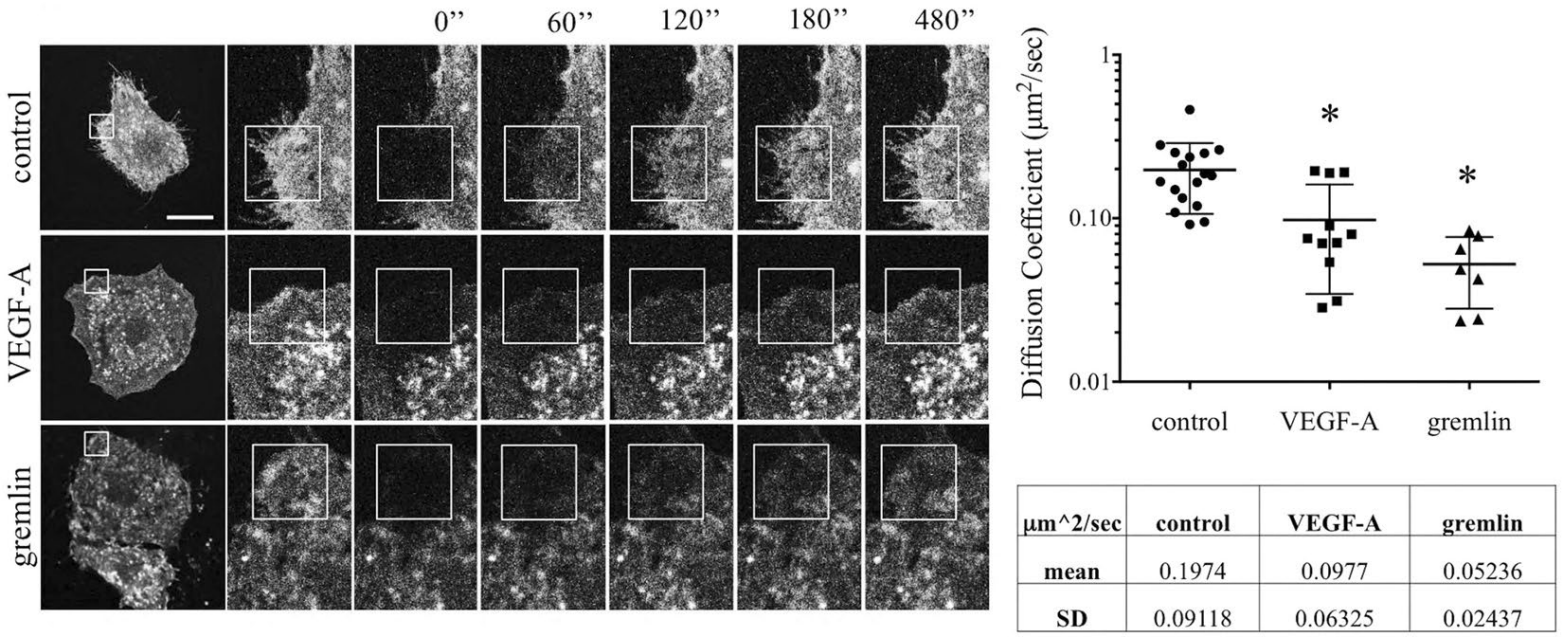
VEGF-A and gremlin reduce VEGFR2 motility on EC surface. (**A**) FRAP analysis was performed on cell plasma membrane of serum-starved ECD-VEGFR2-EYFP over-expressing GM7373 cells treated or not with VEGF-A or gremlin (50 ng/mL). Images were acquired at one per minute for 12 minutes, 2 before and 10 after bleaching. The bleached area is indicated by a square and the recovery time is indicated over the images as seconds after photobleaching (630x; white bar: 20 *μ*m). (**B**) Collected images were analyzed using simFRAP ImageJ plugin to calculate diffusion coefficients. The graph shows diffusion coefficient mean ± s.d. of control, VEGF-A, and gremlin treated cells (*n* = 7 – 15; *P < 0.01, Student’s t-test).

In order to measure the mobility of VEGFR2 on the cell membrane, we performed Fluorescence Recovery After Photobleaching (FRAP) analysis on EC culture expressing ECD-VEGFR2-EYFP. The rate of fluorescence recovery provides quantitative information about the kinetics of diffusion of fluorescent molecule in the photo-bleached area. To measure the dynamics of VEGFR2 on the cell membrane, fluorescence was recorded every minute for 10 minutes in an irreversibly photo-bleached membrane region of ECD-VEGFR2-EYFP EC in the absence or in the presence of 50 ng/mL of VEGF-A or gremlin. In our experimental conditions, 77% of ECD-VEGFR2-EYFP in the plasma membrane turns out to be in a mobile form, with a lateral diffusion coefficient of 0.198 *μ*m^2^/s in untreated ECs. Both VEGF-A or gremlin treatments decrease the receptor mobility respectively to 0.098 *μ*m^2^/s and 0.052 *μ*m^2^/s (Fig. 2). All these data highlight that non-activated receptors are mainly free to move on the cell membrane, thus suggesting that VEGFR2 phosphorylation, its dimerization, and its interaction with membrane co-receptors or intracellular signaler reduce its motility. FRAP data support our former observations^14^ that VEGFR2 is rapidly recruited and immobilized in the membrane in close contact with ligands. These events lead to increase the receptor concentration in the basal side of the cell.

### A chemo-transport-mechanical model describes VEGFR2 relocation on EC surface

**Figure 3:**
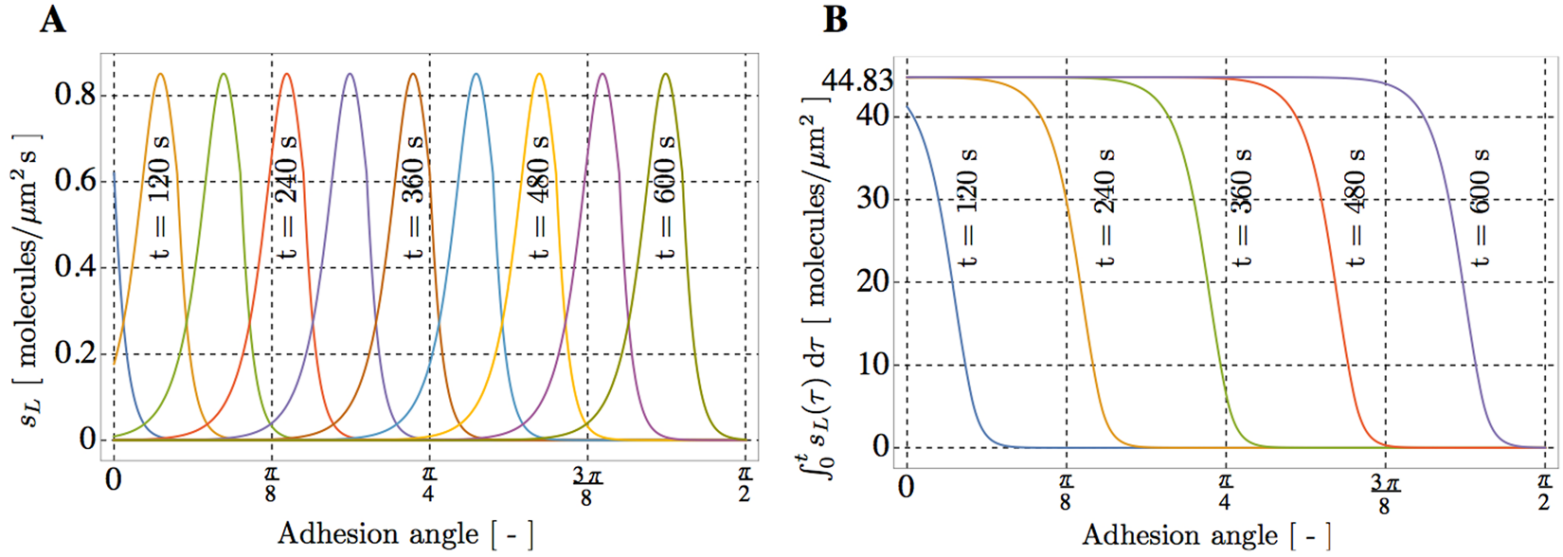
Effects of cell deformation mimicked through a supply of ligands *s*_*L*_ onto the membrane. (**A**) Spatial evolution of the mass supply *s*_*L*_ and (**B**) of its time-cumulate.

We describe the relocation of VEGFR2 on the cell membrane during its adhesion to ligand-enriched ECM by means of a model that accounts for the ligands-receptors chemical interaction, the diffusion of receptors along the membrane, and the mechanical deformation of the cell. It will be denoted henceforth as a chemo-transport-mechanical model. It is defined on the cell surface only, and it has been validated against co-designed experimental investigations. The cell adhesion to a ligand-enriched substrate results in VEGFR2 polarization to the basal membrane and entails several concurrent phenomena, including cell deformation and cytoskeletal remodeling, which lead to an increased interaction between the basal cell membrane and the ligand enriched-substrate. While the intracellular tail of VEGFR2 and in particular the tyrosine residues are required for the correct VEGFR2 internalization^29,30^, the kinase domain is not required for the receptor relocation, as demonstrated either by the recruitment of VEGFR2 in the presence of the Sugen 5416^14^ or by the recruitment of the receptor without its intracellular tail (ECD-VEGFR2). VEGFR2 polarization is not mediated by integrin interaction even though, once recruited, VEGFR2 will form an active complex with integrin in lipid rafts. We neglect accordingly the intracellular subset of VEGFR2 and the interaction with coreceptors in our chemo-transport-mechanical model. To simulate the interaction between VEGFR2 and its immobilized ligand, we assume a fixed membrane geometry and account for the effects of cell adhesion with a supply of ligands onto the cell surface at a prescribed rate, *s*_*L*_ (ligands/*μ*m^2^ s), detailed in Eq. (8). Figure 3 shows the spatial evolution of the mass supply *s*_*L*_ and of the total amount of ligands parametrized in time: at each location, ligands smoothly reach the saturation limit of 44.83 ligands/*μ*m^2^ s (Fig. 3B). Owing to this modeling simplification, the actual time-evolving geometry of the membrane becomes relatively unimportant and thus, for maximal simplicity, we analyze it as a circumference of radius l = 20 *μ*m and assume that the time-dependent concentrations depend on the curvilinear coordinate.

The model was implemented in a finite element code as a script in Wolfram Mathematica version 10 and calibrated. The simulations run until the final time *t*_*F*_ = 7200 s at the constant temperature 310.15 K with a substrate-adsorbed ligand concentration of 44.83 ligands/*μ*m^2^ (Fig. 3B).

### Multi-physics drive the complex formation with three different mechanisms

**Figure 4:**
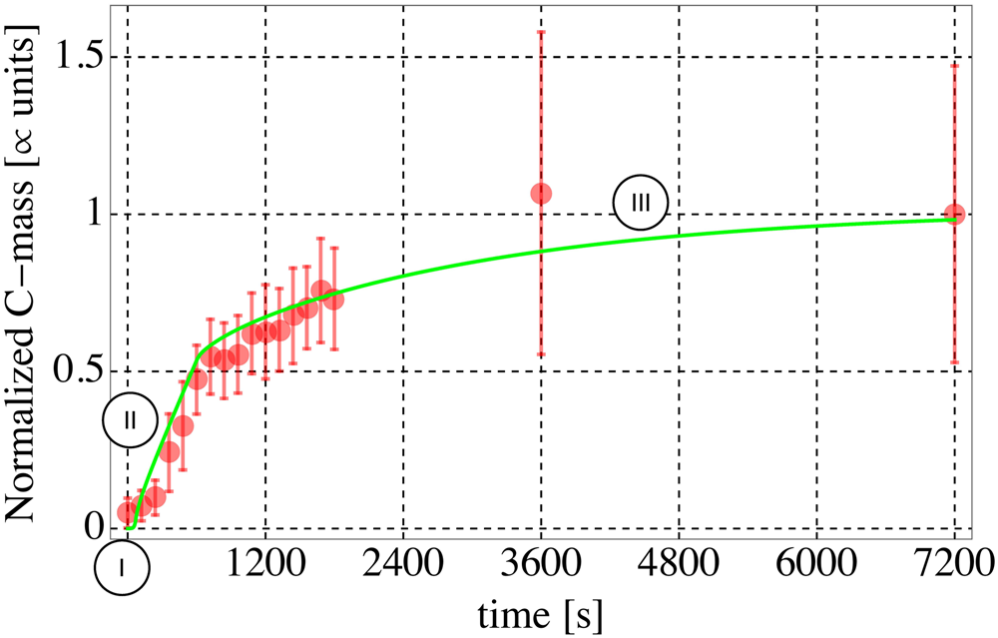
Time evolution of the VEGFR2-gremlin complex formation on the EC membrane. Comparison is made between the VEGFR2 total fluorescence intensity (free and bound) in contact the substrate (red dots) and the numerical simulation data (green lines). To allow comparisons, both sets of data have been normalized to the values reached at the final time *t*_*F*_ = 7200 s.

As depicted in Fig. 4, that represents the overlay of the outcomes of simulation (green line) and *in vitro* experiments (red dots)^14^  normalized to the value of VEGFR2 at the final time *t*_*F*_, VEGFR2 recruitment induced by immobilized ligands shows three phases of complex formation marked by circled roman numbers: an initial plateau (I), a steep branch (II), and finally an evolution with a lower formation rate (III). Our numerical simulations allow connecting these three phases to three distinct mechanisms dominated by different limiting factors. The initial plateau is governed by the cell-ligand contact (I), the second steep phase (that ends at 600 s) is due to a chemo-mechanical evolution - induced by the cell attachment and deformation (II), and the final slow phase reflects the diffusive slow motion of the receptors from the apical to the basal membrane that is in contact with the substrate (III).

The quantitative correspondence between experimental and numerical outcomes suggests that the number of well-oriented ligands available for the receptor binding is much smaller than the total amount of immobilized ligands. As shown in Fig. 4, the simulated evolution in time of the overall amount of bound VEGFR2-ligands complex on the membrane overlaps the experimental outcomes we previously observed^14^, validating of the model.

### The chemo-mechanical transport model describes VEGFR2 dynamics

**Figure 5:**
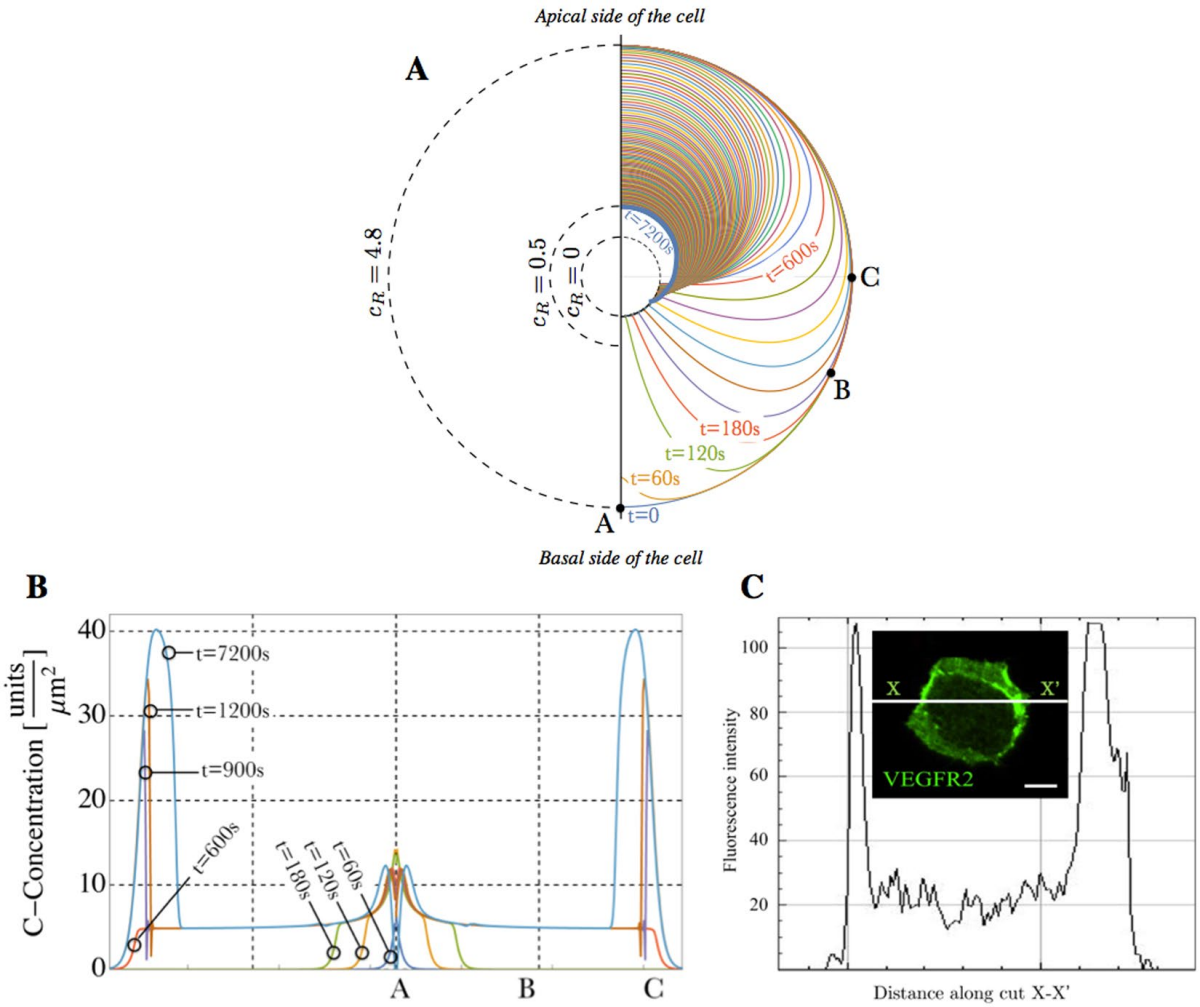
The chemo-mechanical transport model describes VEGFR2 relocation dynamics. (**A**) Time evolution of the spatial concentration *c*_*R*_ of free VEGFR2 along the cell membrane. Each curve plots the distribution of free receptors at different times *t* = 60 *n*, with *n* = 0, 1, 2, … 120 s from the beginning of the experiment at *t* = 0 to the final time *t*_*F*_ = 7200 s. (**B**) Spatial evolution of the concentration *c*_*C*_ of the receptor-ligand complex at various times. The curves report the numerical simulation: points A, B, and C correspond to those in (**A**). (**C**) VPM staining for VEGFR2 confirms peaks in the intensity of fluorescence at the boundary of the substrate/membrane contact surface.

Numerical simulations predict the evolution of the concentration of free receptors (*c*_*R*_) during 2 hours of cell stimulation. Figure 5A quantifies *c*_*R*_ at each location along the membrane at different times. Exploiting the axial symmetry of the simulations, each curve on the right side of Fig. 5A depicts the spatial concentration profile every minute. At *t* = 0 the distribution of receptors is uniform at the concentration *c*_*R*_ = 4.8 receptors/*μ*m^2^. After 60 s, the concentration profile is perturbed and decreases at the bottom of the cell due to receptor-ligand complex formation. As time goes by, starting from 120 s, an enlarging zone with negligible concentration *c*_*R*_ ~ 0 of free receptors is visible at the basal side of the cell (point A), due to the engagement of free receptors by immobilized ligands. At the end of the simulation, at *t*_*F*_ = 7200 s, the concentration of unbound receptors at the apical side amounts at *c*_*R*_ = 0.5 receptors/*μ*m^2^.

The chemo-mechanical transport model allows concluding that the depletion of free receptors is due to three concurrent factors: i) the infinitely fast kinetics of the ligand-receptor interaction; ii) the high equilibrium constant, that favors the formation of ligand-receptor complex; iii) the evidence that diffusion of the receptor on the cell membrane is much slower than interaction kinetics.

The depletion “propagates” with time, so that at *t*_*f*_ = 600 *s*, after the cell is completely adhered, the lower portion of the cell membrane is essentially empty of free receptors. Since no further supply *s*_*L*_ is provided afterwards, the process becomes diffusion-dominated, and it slowly evolves towards a final steady state. The thick blue curve plots the distribution of free receptors at the end of the simulation at time *t*_*F*_. The maximum concentration of free VEGFR2 at *t*_*F*_ is 0.49 receptors/*μ*m^2^ and a steady state has not yet been reached.

### Ligand-receptor complex accumulates at the boundary of the EC basal membrane

Numerical simulations predict that after 2 hours of adhesion (at *t*_*F*_ = 7200 *s*) a zone with high ligand-receptor complex concentration manifests at the boundary of the contact area. Figure 5B depicts the evolution of complex *c*_*C*_ in space (X axis) and time (different colors) at the basal aspect of ECs. Such distribution profile was confirmed experimentally in EC VPMs, as shown in Fig. 5C. VPMs were obtained by an osmotic shock of ECs, that preserves only the basal portion of cell membrane in close contact with ECM, allowing the visualization of the recruited receptors VGEFR2.

## Discussion

VEGFR2 is a transmembrane protein, a key signal transducer in angiogenesis, and a potential therapeutic target in angiogenesis-related diseases. It has a lateral mobility of about 0.198 *μ*m^2^/s, as measured in this work by means of FRAP experiments, and can interact with both soluble and ECM-immobilized ligands. Indeed several VEGFR2 ligands contain one or more heparin binding domains in their sequences. After ligand interaction, VEGFR2 dimerizes and transduces an intracellular signaling via its relocation on the cell membrane and the recruitment of intracellular proteins.

We developed a multi-physics model to describe and predict the effects of ligands on VEGFR2 relocation during the EC activation. The interaction between ligands and receptors has been modeled by a chemical reaction that produces a complex, as customary in literature^19,28^. The model accounts for finite reaction kinetics, although simulations have been carried out assuming that the reaction kinetics is infinitely fast. The time-scale of the VEGFR2-gremlin binding reaction is in fact assumed to be much faster than the time-scale of the mechanical deformation of the cell and of the diffusion of receptors on cell membrane. Under these assumptions, we recovered the experimental evidence that the motion of receptors and their subsequent trapping into immobile VEGFR2-ligands complexes proceed in a sequence of three phases, and we characterized those phases with different rate-controlling factors. The first phase starts when a small portion of membrane gets in contact with the substrate, which provides a sudden supply of ligands that immediately trap the available receptors. This phase is very rapid, because the reaction rate is the controlling factor (in our model assumed infinite), and fully depletes the concentration of free receptors, because the equilibrium constant is very large. The second phase (calibrated in 10 minutes from experiments) is rate-controlled by the mechanical deformation of the cell, which provides additional supply of ligands that afresh immediately react with the available receptors on the newly formed contact area. The mechanical deformation of the cell and the VEGFR2 recruitment^14^ are influenced by the chemical affinity of the VEGFR2-gremlin binding reaction coupled with intracellular cortical actin dynamics. In our co-designed experimental and theoretical study, the cell adhesion is not mediated by integrin engagement, even though integrin involvement cannot be completely ruled out. We observed that in our experimental conditions substrate-immobilized growth factors act as a cell-adhesive stimulus for ECs, which is weaker than the ECM^13^. Although a few papers consider the integrin-mediated focal adhesions and their interactions with the cytoskeleton^31–33^, they do not apply to our experimental conditions since the involved *β*_3_ integrin is not recruited in the focal adhesion complexes. Bound VEGFR2 forms a complex with *β*_3_ integrin in membrane lipid raft domains and not in focal adhesion. Our current simplified model does not capture the complexity of this binding. Instead, it surrogates the details of the mechanical deformation with the experimentally-guided assumption of an empirical *ad*-*hoc* time-sequence of gremlin supply on increasing portions of the cell membrane. The cell surface becomes depleted of free receptors very rapidly where the cell adheres to the substrate. When the mechanical deformation terminates and the cell is eventually spread, the diffusion of receptors becomes the rate-controlling mechanism. During this final phase, receptors that diffuse through the boundary of the contact surface are immediately trapped and immobilized by the ligands on the substrate. Therefore, the VEGFR2-gremlin complex tends to accumulate at the boundary of the basal aspect of the cells in close contact with ECM. Such a localization was observed in the ventral plasma membrane of ECs adherent on gremlin- or HIV-1 Tat^13^ enriched surfaces. A higher concentration of receptors at the cell boundary could have relevant biological implications for the cell, which may sense ligand concentration variation and migrate in the direction of stimulus production.

The key features of our experimental evidence on VEGFR2 relocation are captured well. The effect of the mechanical deformation of the cell has been accounted for in the model, by surrogating the explicit description of the cell spreading/deformation with a given increase in time of the surface area available for the chemical reaction. The model accounts for finite reaction kinetics, although simulations have been carried out assuming that the reaction kinetics is infinitely fast. Moreover, the model shown predictive capability. Numerical simulations highlighted a zone with high complex concentration at the boundary of the contact area at the final time of the simulations. This concentration were experimentally confirmed Fig. 5B and has been understood.

Other authors^19^ proposed meta-models that involve the integration of extracellular cues with intracellular processes, such as receptor endocytosis and phosphorylation. Those models are typically point-wise in nature and focus on the evolution in time disregarding the space distribution, so that ordinary differential equations suffice. Modeling the motion of membrane receptors concurrently with several other aspects of endothelial biology (as internalization or cooperativity in ligand action) is more intricate and can only be dealt with incrementally, moving from simple yet not simplistic assumptions and adding complexities stepwise. The current model will be enriched toward the ambitious target of modeling the angiogenesis process as a whole. The role of kinetic constants will be investigated^34–37^, including internalization^38,39^ as well as the involvement of other co-receptors (including integrins and heparansulphateproteoglycans) and the cytoskeleton re-organization that leads to cell motility and morphogenesis. The interaction between the ligand complex and non engaged integrins will be considered, because *β*_3_ integrin mediates and triggers the long-lasting VEGFR2 activation^14^.

The surrogated mechanics will be replaced by a detailed analysis of the structural behavior of the cell, following recent studies^31–33,40–42^, thus coupling the evolution for the Laplace-Beltrami-like operator that presides the formulation with the large deformation of the cell. Mechanical models for cell spreading involve sophisticated descriptions of active and passive behavior of cells with equations that are much harder to follow than the ones presented here. Simulations of these models require an impressive computational burden, so large that high performance computing is mandatory. In conclusion, there is a substantial increment of theoretical and computational complexity in adding further phenomena of endothelial biology. Since the simplified model with surrogated mechanics resulted capable to reproduce experimental evidences, we expect that the real mechanical evolution may add further insights and fine tune the results, without altering the general picture presented here. A simple yet effective surrogate model may have merits, though, and it will be used to save computational costs when it comes to perform uncertainty quantification studies, which require a large number of computationally expensive simulations.

## Methods

### Cell cultures

Foetal bovine aortic endothelial GM7373 cells^43^ were grown in Dulbecco’s modified Eagle medium (DMEM, Gibco, Life Technologies) containing 10% FCS, vitamins, essential and non-essential amino acids. GM7373 cells were transfected with a pcDNA3/Enhanced Yellow Fluorescent Protein (EYFP) vector harboring the extracellular domain of human VEGFR2 (ECD-VEGFR2) cDNA (provided by K. Ballmer-Hofer, PSI, Villigen, Swiss) to generate ECD-VEGFR2-EYFP EC^14^, or with a pcDNA3.1 vector harboring the mouse VEGFR2 cDNA to generate stable GM7373-VEGFR2 (VEGFR2- ECs).

### Chemotaxis assay

This was performed as described^44^. Briefly, ECD-VEGFR2-EYFP overexpressing ECs were seeded at 3.0 × 10^6^ cells/mL in *μ*-slide chemotaxis chambers (IBIDI, Martinsried, Germany) and incubated in complete medium for 24 hours. Cells were then stimulated with gremlin^11^ and VEGF-A (100 ng/mL)^45^ and observed during gradient formation. After 2 hours, cells were photographed under a Zeiss Axiovert 200 M epifluorescence microscope equipped with a Plan-Apochromat 63x/1.4 NA oil objective. Z-stack images acquired using ApoTome imaging system were elaborated through AxioVision Extended Focus module (Zeiss Axiovert 200 M system).

### ECs adhesion on immobilized protein

2.0 *μ*g/mL of gremlin, VEGF or Fibrinogen (Fb) were added to polystyrene tissue culture plates or glass coverslips. After 16 hours of incubation at 4 °C, samples were washed and uncovered glass was blocked with 1.0 mg/mL bovine serum albumin (BSA) for 1 hour at room temperature^14^.

### Ventral plasma membrane (VPM) preparation

VPMs were prepared by osmotic shock using a modification of the squirting lysis technique^14^. Briefly, cells were washed twice with ice-cold water; after 1 minute cells were squirted over by using a jet of ice-cold water and immediately fixed for immunocytochemistry analysis. In all the experiments, the absence of DAPI staining and the persistence of actin filaments were used to unequivocally identify the VPM remnants bound to the substratum. For total VEGFR2 staining, samples were incubated overnight with a rabbit polyclonal anti-VEGFR2 antibody (1:200, Santa Cruz Biotechnology) followed by a 1-hour incubation with AlexaFluor 488-conjugated anti-rabbit IgG (1:500). For actin staining samples were incubated for 30 minutes with TRITC-phalloidin (0.9 mg/mL in PBS, Sigma). All reagent dilutions were in PBS containing 3% BSA. VPMs were acquired under an Axiovert 200 fluorescence microscope equipped with a Plan-Apochromat 63x/1.4 NA oil objective and ApoTome system (Carl Zeiss). VEGFR2-positive areas and total VPM areas, defined by actin staining, were quantified using Image-Pro Plus software.

### Ligands/cell binding assay

GM7373-VEGFR2 (VEGFR2-ECs) cells were seeded on VEGF-A or gremlin or uncoated coverslip for 4 hours and then incubated with soluble gremlin (150 ng/mL) for 90 minutes at 4 °C in PBS added with Ca2+ and Mg2+ and 0.1% gelatin. Then, cells were washed three times with PBS or with PBS plus 1.5 mol/L NaCl to remove HSPGs-bound ligands. Immunofluorescence analysis was performed using a goat polyclonal anti-gremlin antibody (R&D Systems) or goat polyclonal anti VEGF-A antibody followed by AlexaFluor 488 anti-goat IgG (Molecular Probes, Life Technologies). Cells were analysed using a Zeiss Axiovert 200 M system.

### Fluoresence Recovery After Photobleaching (FRAP)

ECD-VEGFR2-EYFP ECs were seeded at 5.0 × 10^5^ cells/mL *μ*-slides (IBIDI, Martinsried, Germany). Cells were starved in minimal medium for 4 hours, stimulated with gremlin or VEGF-A (50 ng/mL) and analysed using LSM510-META confocal microscope equipped with an incubation chamber (Zeiss). Confocal images were recorded with 4–5% of the intensity of the 514-nm line from living transfected cells. EYFP fluorescence was eliminated using a 350-iteration bleach cycle at 100% intensity of the 514-nm line. Control bleach experiments performed over the entire cell surface demonstrated that the EYFP chromophore was completely inactivated by this treatment and that recovery of fluorescence due to newly synthesized EYFP proteins was not detectable during the period of recovery (10 minutes). Image series were analysed using simFRAP ImageJ plugin (https://imagej.nih.gov/ij/plugins/sim-frap/index.html)^46^. To calculate diffusion coefficients and using FRAP Caculator ImageJ macro (https://www.med.unc.edu/microscopy/resources/imagej-plugins-and-macros/frap-calculator-macro) to calculate mobile and immobile fractions following author instructions.

### Data representation

Data are expressed as mean ± SD or mean ± SEM. Statistical analyses were performed using the Student’s t-test. The significance level was set at P < 0.01.

### Chemo-transport-mechanical model formulation

A general formulation for the chemo-transport-mechanics problem with trapping is here tailored to model the relocation of VEGFR2 on the lipid bilayer membrane. The interaction between receptors (*R*) and ligands (*L*) is described as a chemical reaction, which produces a receptor-ligand complex (*C*),

1 $$R+L\underset{{k}_{1}^{-}}{\overset{{k}_{1}^{+}}{\rightleftarrows }}C.$$

The ligand, whose degradation is negligible, and the complex are assumed to be immobile. Complex internalization and the return back to the surface will be elaborated in future publications. Since receptors are free to move along the membrane, reaction (1) portrays a conversion of mobile to trapped receptors and vice-versa. Its rate is denoted with *w* ^(1)^ (molecules/*μ*m^2^ s). Therefore, the mass balance equations for the three species involved in reaction (1) read:

2a $$\frac{\partial {c}_{R}}{\partial t}+{\rm{div}}\,[{\overrightarrow{h}}_{R}]+{w}^{(1)}=0,$$

2b $$\frac{\partial {c}_{L}}{\partial t}+{w}^{\mathrm{(1)}}={s}_{L},$$

2c $$\frac{\partial {c}_{C}}{\partial t}-{w}^{\mathrm{(1)}}=0.$$

Since Eq. (2) are defined on the cell membrane only, derivatives in the divergence operator are defined on a curved surface, in the Laplace-Beltrami sense. Concentrations *c*_*R*_, *c*_*L*_, *c*_*C*_ (molecules/*μ*m^2^) are constrained fields, since they must be positive and cannot exceed the saturation amounts ${c}_{R}^{{\rm{\max }}}$, ${c}_{L}^{{\rm{\max }}}$, ${c}_{C}^{{\rm{\max }}}$, respectively. Symbol *s*_*L*_ denotes a mass supply (molecules/*μ*m^2^ s), which will be illustrated later in the paper. Vector ${\overrightarrow{h}}_{R}$ (molecules/s*μ*m) denotes the flux of receptors along the membrane surface. It is constitutively described by a Fickian law, by means of the tangential gradient of the concentration,

3 $${\overrightarrow{h}}_{R}=-{{\rm{D}}}_{R}\,\nabla \,[{c}_{R}],$$

thus entailing the Clausius-Duhem inequality. Symbol ${{\rm{D}}}_{R}$ denotes the receptors’ diffusivity along the membrane (*μ*m^2^/s). The chemical kinetics of reaction (1) is modeled via the law of mass action^47^,

4 $${w}^{\mathrm{(1)}}={k}_{1}^{+}\frac{{\theta }_{L}}{1-{\theta }_{L}}\frac{{\theta }_{R}}{1-{\theta }_{R}}-{k}_{1}^{-}\frac{{\theta }_{C}}{1-{\theta }_{C}},$$

where ${k}_{1}^{\pm }$ denote the forward (+) and reverse (−) rate constants (molecules/*μ*m^2^ s) for reaction (1), ${\theta }_{J}={c}_{J}/{c}_{J}^{{\rm{\max }}}$ for *J* = *R*, *L*, *C*, and the chemical potentials are assumed of the form

$${\mu }_{J}={\mu }_{J}^{0}+RT\,\mathrm{ln}\,\frac{{\theta }_{J}}{1-{\theta }_{J}},$$

with each ${\mu }_{J}^{0}$ independent of all the concentrations. At chemical equilibrium the concentrations obey the relation

5 $$\frac{{\theta }_{C}^{{\rm{eq}}}}{1-{\theta }_{C}^{{\rm{eq}}}}\frac{1-{\theta }_{L}^{{\rm{eq}}}}{{\theta }_{L}^{{\rm{eq}}}}\frac{1-{\theta }_{R}^{{\rm{eq}}}}{{\theta }_{R}^{{\rm{eq}}}}={K}_{{\rm{eq}}}^{\mathrm{(1)}}=\exp [-\frac{{\rm{\Delta }}{G}_{\mathrm{(1)}}^{0}}{RT}],$$

where ${K}_{{\rm{eq}}}^{\mathrm{(1)}}$ is the equilibrium constant and ${\rm{\Delta }}{G}_{\mathrm{(1)}}^{0}={\mu }_{C}^{0}-{\mu }_{R}^{0}-{\mu }_{L}^{0}$ is the Gibbs free energy of the reaction.

Mass balance equation (2) shall be accompanied by the balance of force, to model the mechanical (and thus geometrical) evolution of the cell shape, whose boundary - the membrane - is the geometrical support of Eq. (2). Modeling the evolution of the Laplace-Beltrami operator that presides formulation (2–4) concurrently with the large deformation of the cell is a phenomenally ambitious task, which is in progress motivated by the promising outcomes here shown. In the present work, we surrogate the mechanics with some simplifying assumptions. They are collected in the following section.

### Simplifying assumptions

Two major simplifications, pertaining to reaction kinetics and mechanical modeling, will be undertaken henceforth. They allow verifying the capability of the chemo-transport model (2–4) to reproduce experimental evidences with much less theoretical and computational burden.

The assumption is taken henceforth that the reaction kinetics is infinitely fast, considering that the time required to reach chemical equilibrium is orders of magnitude smaller than the time-scale of the other processes. This assumption is usually taken in conceptually similar problems, as the diffusion of species in metals^48^ or the electro-mechanics of batteries^49–53^. Then, concentration of the complex is governed by thermodynamic equilibrium *at all times* by Eq. (5), which with trivial algebra becomes:

6 $${c}_{C}({c}_{L},{c}_{R})={c}_{C}^{{\rm{\max }}}\,{\{1+\frac{1-{\theta }_{L}}{{\theta }_{L}}\frac{1-{\theta }_{R}}{{\theta }_{R}}\frac{1}{{K}_{{\rm{eq}}}^{\mathrm{(1)}}}\}}^{-1}.$$

The two final parabolic governing equations in the unknown fields *c*_*L*_ and *c*_*R*_ can be easily inferred from Eqs (2a) and (2b), by replacing (2c) with (6)

7a $$\frac{\partial {c}_{R}}{\partial t}+\frac{\partial {c}_{C}({c}_{L},{c}_{R})}{\partial t}+{\rm{div}}\,[{\overrightarrow{h}}_{R}]=0,$$

7b $$\frac{\partial {c}_{L}}{\partial t}+\frac{\partial {c}_{C}({c}_{L},{c}_{R})}{\partial t}={s}_{L}\mathrm{.}$$

The change of the geometry of the membrane with time is dictated by the mechanical evolution of the cell, which in turn is governed by several complex phenomena (such as focal adhesion and stress-fiber reorganization^32^, integrins-ECM interactions^54^, curvature changes of cell membrane^55^, cooperation between integrins and VEGFR2^45,56^) triggered by the adhesion of the cell onto the ligand-coated plate described in the experimental section. Roughly, adhesion induces cells to deform from an initially spherical shape to a final spread configuration^33,57^, resulting in increased interaction between basal cell membrane and the ligand enriched-substrate. We replace the explicit modeling of cell spreading/deformation with a supply of ligands *s*_*L*_, calibrated from experimental data and included in Eq. (7) in order to simulate the interaction between basal cell membrane and the substrate.

We assume therefore that the membrane geometry is fixed and thus *account for the effects* of cell adhesion by a supply of gremlin on the membrane at a prescribed rate, *s*_*L*_ (molecules/*μ*m^2^ s), inferred from experimental evidence and defined as follows:

8 $${s}_{L}(x,t)=\frac{\overline{{c}_{L}}}{\overline{t}}\, {\mathcal H} \,[t-\frac{x}{v}]\,\, {\mathcal H} \,[\overline{t}-t+\frac{x}{v}]$$

and plot in Fig. 3. In Eq. (8) ${\mathcal H}$ is the Heaviside step function, $\overline{{c}_{L}}=72\,{\rm{ligands}}/\mu {{\rm{m}}}^{2}$ is the concentration of substrate-immobilized ligand available for reaction (1), *t*_*f*_ is the time required for the complete mechanical deformation of the cell, $v=\pi \ell /2{t}_{f}$ is the velocity of mechanical deformation (assumed to be constant until *t*_*f*_), $\ell$ is the cell radius, $\overline{t}\ll {t}_{f}$ is a parameter that identifies a finite time required for binding, *x* is the curvilinear abscissa of our simplified geometry, *t* the generic time. In view of Eq. (8), the supply of ligands at point *x* on the membrane remains zero until *t* < *x*/*v*; then, in the time span between *t* = *x*/*v* and $t=x/v+\overline{t}$, it increases rapidly from zero to $\overline{{c}_{L}}$.

### Weak form and discretization

The governing equations for the relocation of VEGFR2 on the membrane under the above modeling assumptions have been made dimensionless and multiplied by test functions. The weak form obtained by their integration over the spatial domain can be transformed to a first order Ordinary Differential Equation (ODE) in time if the discretization is performed via separated variables. Therefore, nodal unknowns depend solely on time, while test and shape functions solely on space. Time advancing has been achieved by finite differences, using a backward Euler scheme. Discretization of the unknown fields by means of standard linear shape functions leads to the numerical approximation via the finite element method in each time step.

### Material parameters

Parameters for the in silico simulation (see Table 1) were defined by *in vitro* assays. The cell radius was calculated from the measure of radius of 50 ECs using Zeiss Axiovert 200 M microscope; receptor diffusivity was obtained by FRAP analysis (see above). The amount of VEGFR2 on cell membrane per area was calculated by dividing the number of high affinity binding sites, obtained by radiolabeled binding experiments^58^ for cell surface area. The kinetic parameters ligand/receptor were measured by Surface plasmon resonance (SPR) measurements (BIAcore X, GE Healthcare). The extracellular domain of human VEGFR2 was immobilized onto CM5 sensorchips (BIAcore) and increasing concentrations (from 100 ng/mL to 4 *μ*g/mL) of ligand was injected in HBS-EP buffer (BIAcore) for 10 minutes (sample volume: 50 *μ*L; flow rate: 5 *μ*L/minute; dissociation time: 2 minutes). Binding parameters were calculated by the nonlinear-curve-fitting software package BIAevaluation 3.2 (Biacore)^58^. The maximal surface density of immobilized ligand on surface was determined by SPR. The sensorgram recorded during immobilization gives direct check on the amount of ligand attached to the surface. Full details and references about this procedure can be found at the dedicated page of the SPR pages website: http://www.sprpages.nl/Experiments/Howmuch.php.

**Table 1:** Material parameters used in the simulations and their bibliographic source.

*Notation*	*Value*	*Units*	*Ref*.	*Notation*	*Value*	*Units*	*Ref*.
$\ell$	20	*μ*m	this study	${K}_{{\rm{eq}}}^{\mathrm{(1)}}$	354059	—	59
${{\rm{D}}}_{R}$	0.198	$\frac{\mu {{\rm{m}}}^{2}}{s}$	this study	${c}_{L}^{{\rm{\max }}}$	16000	$\frac{{\rm{molecules}}}{\mu {{\rm{m}}}^{2}}$	this study
${c}_{R}^{0}$	4.8	$\frac{{\rm{molecules}}}{\mu {{\rm{m}}}^{2}}$	60	${c}_{R}^{max}$	${c}_{C}^{max}$	$\frac{{\rm{molecules}}}{\mu {{\rm{m}}}^{2}}$	this study
${c}_{L}^{0}$	0	$\frac{{\rm{molecules}}}{\mu {{\rm{m}}}^{2}}$	this study				

### Model calibration

Analyzing the evolution in time of VEGFR2 recruitment induced by immobilized ligands experimentally measured in^14^ as fluorescence intensity of the overall VEGFR2 (free and bound), three phases of complex formation were identified, namely: an initial plateau, lasting few seconds; a steep branch, that takes place until about 600 s; and finally an evolution with a lower formation rate after the first 600 s. Accordingly, we calibrated *t*_*f*_ = 600 s as the time for completion of the mechanical deformation and $\overline{t}=1s$ as the parameter that identifies a finite time required for binding.
